# An Improved Immunochromatographic Strip Based on Plant-Derived E2 for Detection of Antibodies against Classical Swine Fever Virus

**DOI:** 10.1128/spectrum.01050-22

**Published:** 2022-07-12

**Authors:** Qianru Xu, Yaning Sun, Jifei Yang, Fanshu Ma, Yanan Wang, Shenli Zhang, Xueyang Li, Xiaotian Qu, Yilin Bai, Rui Jia, Li Wang, Erqin Zhang, Gaiping Zhang

**Affiliations:** a International Joint Research Center of National Animal Immunology, College of Veterinary Medicine, Henan Agriculture University, Zhengzhou, China; b School of basic medical sciences, Henan University, Kaifeng, China; c Henan Provincial Key Laboratory of Animal Immunology, Henan Academy of Agricultural Sciences, Zhengzhou, China; d CAS Key Laboratory of Nano-Bio Interface, Suzhou Institute of Nano-Tech and Nano-Bionics, Chinese Academy of Sciences, Suzhou, China; e School of Advanced Agricultural Sciences, Peking University, Beijing, China; f Jiangsu Co-Innovation Center for the Prevention and Control of Important Animal Infectious Diseases and Zoonoses, Yangzhou University, Yangzhou, China; Changchun Veterinary Research Institute

**Keywords:** classical swine fever virus, plant-derived E2, immunochromatographic strip, E2 antibodies

## Abstract

Vaccination is an effective method to control the spread of classical swine fever virus (CSFV), which is a major cause of economic losses to the swine industry. Although serological detection assays are commonly used to assess immune status, current methods for monitoring of antibodies (Abs) are time-consuming, expensive, and require cell culture and virus manipulation. To address these problems, the E2 protein of CSFV was expressed in transgenic rice seeds as a labeled antigen for the development of an immunochromatographic test strip (ICTS) for rapid, precise, and cost-effective detection of Abs. The ICTS has a reasonable sensitivity of 1:128,000 for detection of serum Abs against CSFV and no cross-reactivity with Abs of other porcine viruses. The similarity of the results between the proposed ICTS and a commercial enzyme-linked immunosorbent assay was 94.1% (128/136) for detection of serum Abs from immunized animals and 92.3% (72/78) for detection of maternally derived Abs. The proposed assay was successfully used to monitor Abs against E2 of both pigs and rabbits immunized with a live attenuated vaccine or an E2 subunit vaccine. The results confirmed that the ICTS can be applied to detect Ab levels in animals with different immunological backgrounds. The ICTS based on plant-derived E2 is a relatively inexpensive, rapid, and accurate assay for detection of Abs against CSFV and avoids the risk of contamination by animal products.

**IMPORTANCE** The E2 protein of classical swine fever virus (CSFV) was expressed in transgenic rice endosperms as a diagnostic antigen for use with a rapid colloidal gold assay for the detection of antibodies (Abs) against CSFV. This improved test was used to monitor Abs against the E2 protein in both pigs and rabbits immunized with a live attenuated vaccine or E2 subunit vaccine. The assay successfully detected Ab levels in serum samples from piglets with different immunological backgrounds. In contrast to current E2 protein-based diagnostic methods using Escherichia coli or insect cells as expression systems, plant-derived E2 avoids the limitations of low immunogenicity of eukaryotic expression systems and potential contamination of fetal bovine serum with bovine viral diarrhea virus in cell culture.

## INTRODUCTION

Classical swine fever (CSF) is an acute highly contagious viral disease caused by infection with classical swine fever virus (CSFV) that has resulted in significant mortality to domestic and wild pigs worldwide, as well as substantial economic losses to the swine industry (1–3). Biosafety measures and vaccination with both traditional live attenuated vaccines (LAVs) and subunit vaccines have been utilized to control and eradicate CSFV. However, moderately virulent and attenuated CSFV strains cause persistent recessive infection and immunosuppression in the herd, as well as low immune efficacy of vaccines in the presence of maternally derived antibodies (MDAs) in piglets, thereby hindering control of CSFV, which remains endemic in South and Central America, Asia, the Caribbean, and Eastern Europe (4). Furthermore, the re-emergence of CSFV in Japan has demonstrated the continued risk of CSFV (5). Thus, in addition to implementation of enhanced biosecurity measures and the development of more effective vaccines, early clinical diagnosis and surveillance programs are needed for better control of CSFV.

CSFV is an enveloped single-stranded RNA virus belonging to the *Pestivirus* genus and *Flaviviridae* family. Pestiviruses also include bovine viral diarrhea virus (BVDV) and border disease virus (BDV) (6). The CSFV genome consists of approximately 12.3 kb and a single large open reading frame that encodes four structural proteins (C, E^rns^, E1, and E2) and eight nonstructural proteins (N^pro^, p7, NS2, NS3, NS4A, NS4B, NS5A, and NS5B) (7, 8). E2 is the primary protective protein that elicits neutralizing antibodies (Abs) and is the target antigen of molecular and serological diagnostic assays for testing of vaccines against CSFV (9, 10).

Various marker vaccines and several subunit vaccines against CSFV have been developed based on the CSFV E2 protein (11). Appropriate serological Ab detection via large-scale screening is necessary to assess vaccine immunization of pigs against CSFV (12). Furthermore, conventional LAVs provide high levels of MDAs for piglets, including major Abs against the E2 protein. MDAs are considered to protect piglets against field strains of CSFV in the first few weeks after birth (13). However, MDAs are reported to interfere with live vaccines (14). Hence, an evaluation of Abs against the E2 protein would help to determine the earliest time point when MDAs are no longer detectable, rationalize the immunization procedure, improve the efficiency of vaccine immunization, and reveal the introduction of CSFV in a region. Virus neutralization tests and commercial enzyme-linked immunosorbent assays (ELISAs) are both highly sensitive and specific for detection of immune responses. However, these methods are limited by time, cost, and the requirements for cell culture and virus manipulation. Compared to traditional immunoassays, an immunochromatographic test strip (ICTS) offers the advantages of low cost, rapidity, high specificity, high sensitivity, and ease of handling. ICTSs have been previously prepared with E2 antigens generated from Escherichia coli or baculovirus expression systems (15, 16). However, available E2 proteins either have low antigenicity or are cellularly produced; thus, the risk of infection by other pestiviruses, particularly BVDV and BDV, cannot be excluded, implying that improvement is needed for the detection of Abs against CSFV.

Plants have been used as efficient “biofactories” that provide many advantages over traditional fermenting systems for production of recombinant proteins as vaccines, Abs, and bioactive peptides (17, 18). In particular, recombinant proteins produced in plant seeds are very stable at room temperature (RT) without loss of biological activity while facilitating rapid production at low cost without those problems of human and animal pathogens associated with the use of mammalian cells (19). Therefore, the use of plant-derived products can improve the effectiveness of diagnostic reagents. Hence, the aim of the present study was to develop an accurate and sensitive method for detection of CSFV Abs. A recombinant E2 (rE2) protein was produced in rice endosperm, and an immunochromatographic strip was designed for better assessment of vaccine immunization and interference of MDAs.

## RESULTS

### Production and identification of the rE2 protein.

**FIG 1:**
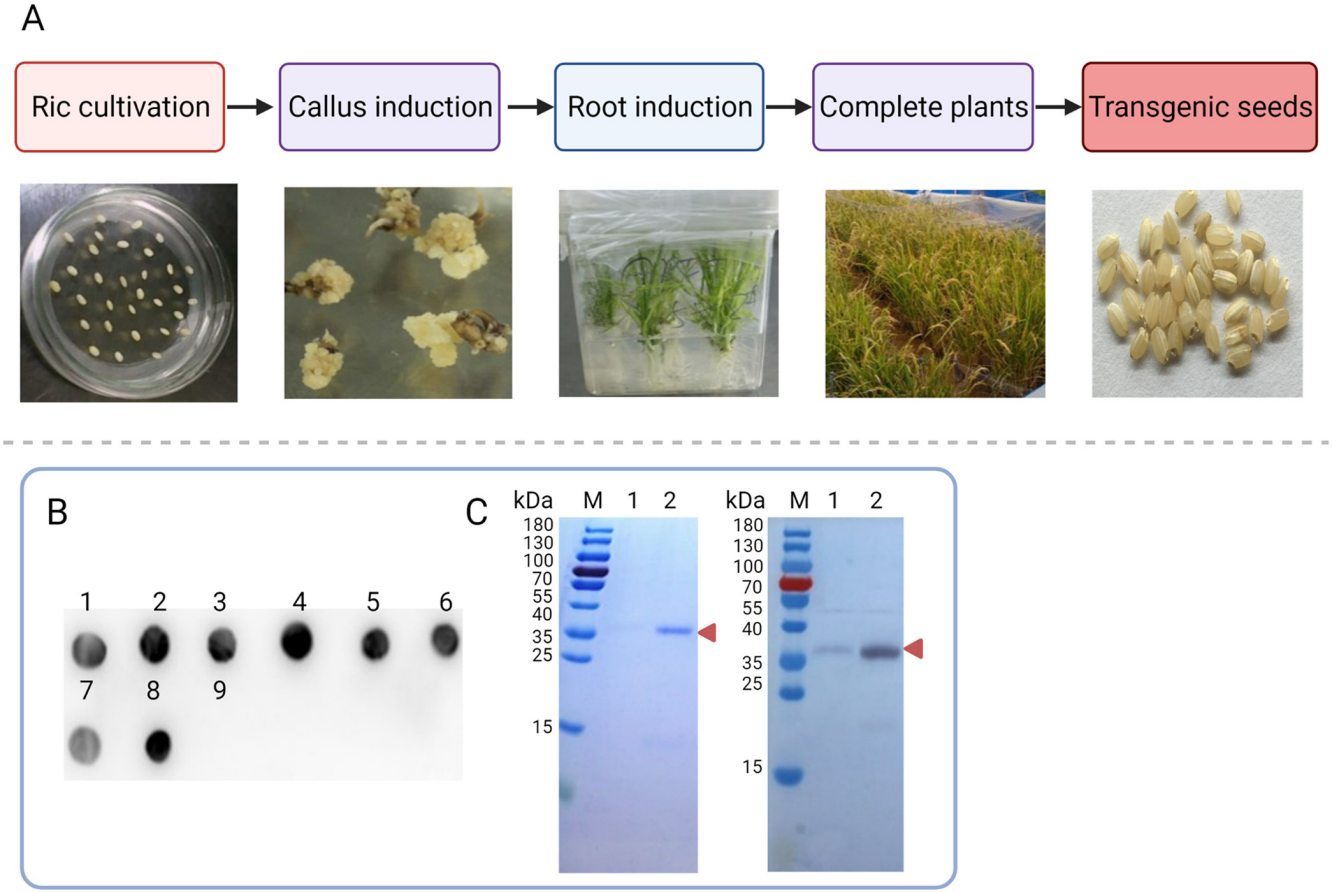
Production and identification of the rE2 protein in rice endosperm. (A) Genetic transformation of rice includes rice cultivation, callus induction, root induction, harvest of intact plants, and collection of mature transgenic rice. (B) Screening of rice plants expressing rE2 by dot blot analysis. Dots 1 to 8 are eight different kinds of transgenic plants. Dot 9 is a TP309 nontransgenic plant (TP309). (C) SDS-PAGE (left) and Western blotting (right) analysis of the purified rE2 protein. M, protein marker. Columns 1 and 2, wash-off fractions. The red arrowhead indicates the rE2 protein.

The rE2 protein was successfully expressed in transgenic rice endosperm (Fig. 1A), and positive rE2-expressing rice plants were screened by dot blot analysis (Fig. 1B). After purification, the concentration of the rE2 protein in buffer (10 mM Tris-HCl [pH 7.5], 25 mM NaCl) was 2.0 mg/mL. Sodium dodecyl sulfate-polyacrylamide gel electrophoresis (SDS-PAGE) determined that the molecular weight of the rE2 protein was ~36 kDa. Western blot analysis showed that the rE2 protein had good reactivity and specificity with CSFV-positive swine serum (Fig. 1C).

### Results with colloidal gold rE2-based ICTSs.

**FIG 2:**
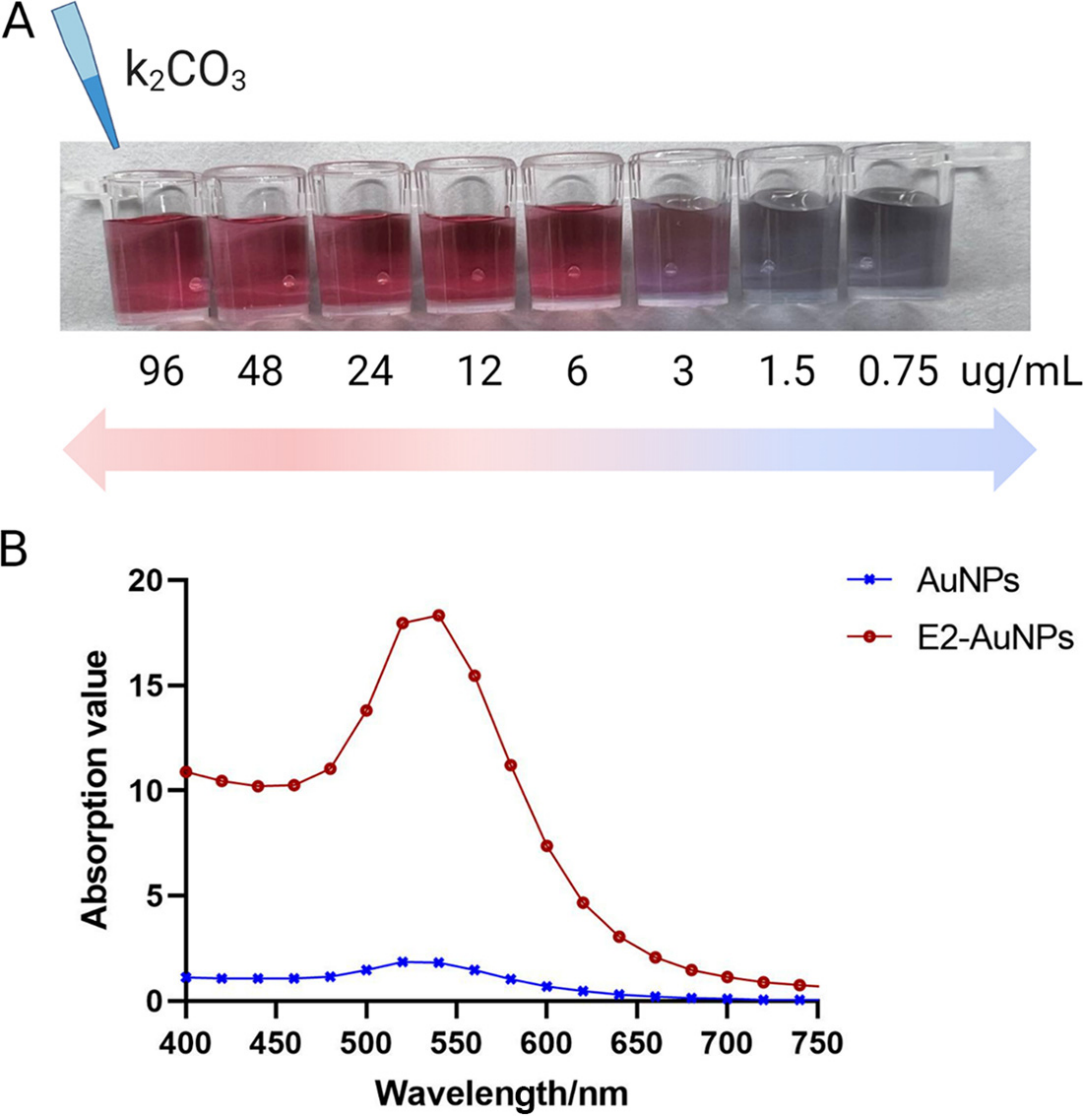
Optimization of the colloidal gold solution. (A) Optimal amount of the rE2 protein. The color of the mixed solution changed from red to dark violet. The optimal concentration of the stable colloidal gold-labeled rE2 protein was 6 μg/mL. (B) UV scanning peaks. The maximum absorption peak of E2-AuNPs was 540 nm. AuNPs, gold nanoparticles; E2-AuNPs, colloidal gold-labeled rE2.

The protein buffer, protein concentration, and pH of the gold suspension buffer were optimized for use with ICTSs for detection of the rE2 protein. Comparisons of different protein buffer systems revealed that the colloidal gold solution prepared with the rE2 protein dissolved in phosphate-buffered saline (PBS) was stable and did not cause precipitation; thus, PBS was the optimal buffer. To detect the optimal amount of the rE2 protein, 125 μL of colloidal gold solution was added to 10 μL of diluted rE2 protein. After 5 min, 125 μL of a 10% NaCl solution was added to verify the destructive test. As shown in Fig. 2A, the color of the mixture changed from red to dark violet and the optimal concentration of colloidal gold-labeled rE2 protein was 6 μg/mL. To optimize the gold suspension buffer, the pH value of the most adhesive binding protein on the surface of the gold particles was screened by adding 2 to 8 μL of 0.2 M K_2_CO_3_ (pH 6.0 to 9.0). The optimal pH for the E2 protein was pH 7.5 to 8.0. After conjugation, the wavelength of colloidal gold-labeled rE2 particles was near 550 nm (Fig. 2B). The optimal conditions for the ICTSs are shown in Table 1.

**TABLE 1:** Optimal conditions for the ICTSs

Condition	Result
Protein buffer	PBS
Protein concn	6 μg/mL
Optimal pH	7.5–8.0
Vol of colloidal gold-labeled rE2	6 μL/cm
Concn of SPA	0.5 mg/mL
Concn of anti-IgG	1 mg/mL

### Specificity, sensitivity, and stability of the rE2-based ICTSs.

**FIG 3:**
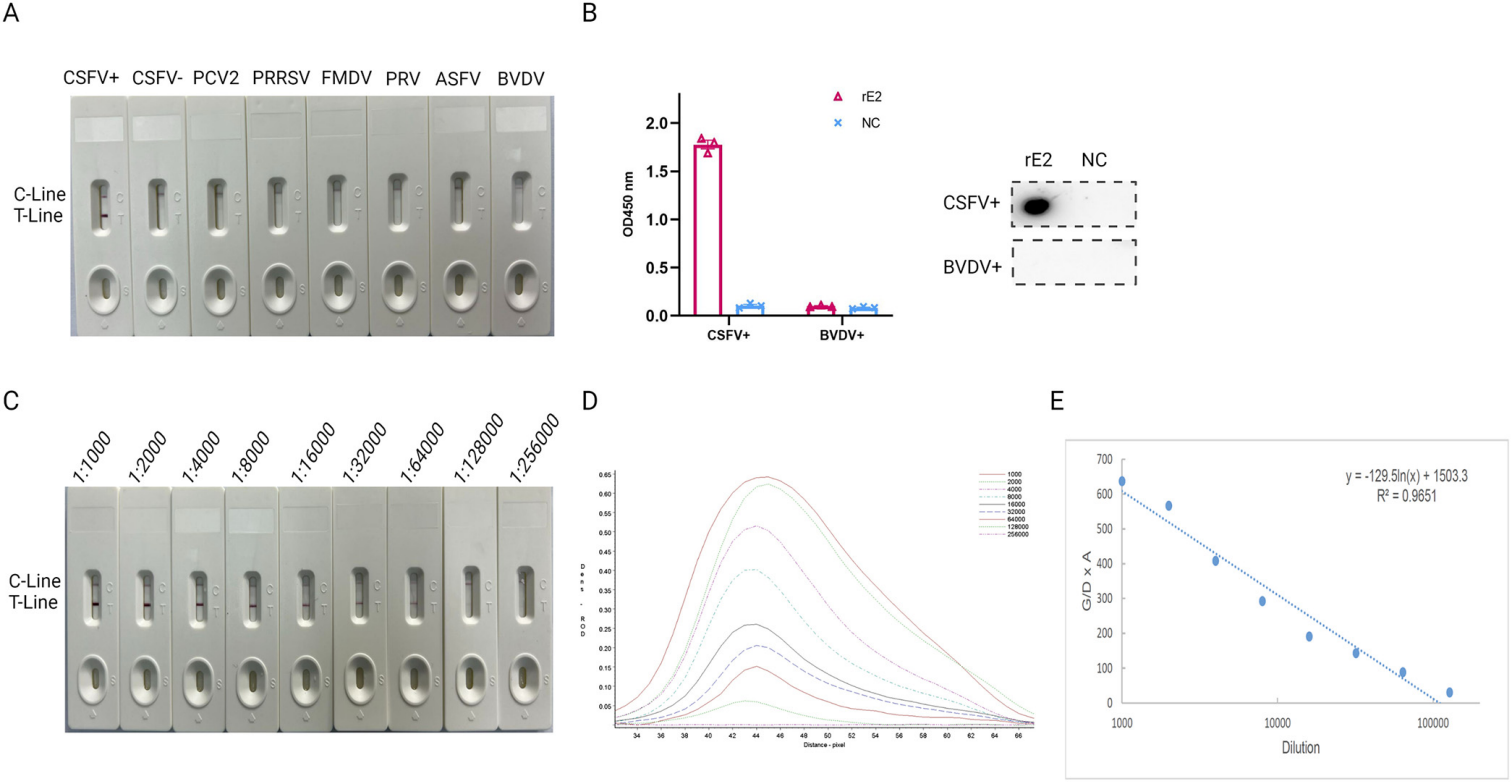
Specificity and sensitivity of rE2-based ICTSs. (A) Specificity of the ICTSs. Seven different serum samples with pathogenic pig viruses were evaluated. No cross-reactivity was detected with the ICTSs. (B) Reactivity of rE2 with CSFV-positive serum and BVDV-positive serum was measured by ELISA (upper) and dot blotting (lower). CSFV+, CSFV-positive serum; BVDV+, BVDV-positive serum; NC, the extraction of soluble protein from TP309. (C) Sensitivity of the rE2-based ICTSs. The CSFV-positive serum was diluted from 1:1,000 to 1:256,000 and tested. C, control line; T, test line. (D) Relative optical density curves of the chromogenic results for the rE2-based ICTSs. (E) Standard curve between the G/D × *A* value and dilution of E2-positive serum.

The specificity of the rE2-based ICTSs was evaluated using a panel of sera against seven different pathogenic porcine viruses, which included CSFV-positive serum (*n* = 10), CSFV-negative serum (*n* = 10), porcine reproductive and respiratory syndrome virus (PRRSV)-positive serum (*n* = 4), porcine circovirus type 2 (PCV2)-positive serum (*n* = 4), foot and mouth disease virus (FMDV)-positive serum (*n* = 4), BVDV-positive serum (*n* = 3), and African swine fever virus (ASFV)-positive serum (*n* = 3). The test results showed that only CSFV-positive serum was positive, with two visible red bands at both the test and control lines, while the other serum samples showed no cross-reactivity (Fig. 3A and Table 2). In addition, the ELISA and dot blotting results verified that the rE2 protein did not cross-react with BVDV-positive serum (Fig. 3B).

**TABLE 2:** Specificity and accuracy

Sample	No. of samples	No. positive	
		ICTS	IDEXX^*a*^
CSFV-positive serum	10	10	10
CSFV-negative serum	10	0	0
PRRSV serum	4	0	0
PCV2 serum	4	0	0
FMDV serum	4	0	0
BVDV serum	3	0	0
ASFV serum	3	0	0
Clinical swine serum	136	128	136
Piglet serum	78	72	78

a Note that the IDEXX CSFV antibody test kit was positive when the blocking rate was >40%.

The sensitivity of the rE2-based ICTSs was further evaluated with CSFV-positive serum serially diluted from 1:1,000 to 1:256,000. Values above the threshold were obtained up to a dilution of 1:128,000, confirming that the assay was highly sensitive (Fig. 3C). Test line measurements using the TSR3000 membrane strip reader showed that (G/D) × A (graph density × area) – relative optical density (ROD) decreased as the standard sample dilution increased (Fig. 3D). The respective regression equation was *y* = −129.5ln(*x*) + 1503.3 (*R*^2^ = 0.9651) (Fig. 3E).

In order to validate the stability of the test, strongly positive, weakly positive, and negative anti-CSFV serum samples were tested with the rE2-based ICTSs at 0, 1, 3, 6, 9, and 12 months. As shown in Table 3, the rE2-based ICTSs can be stored in a dry place at RT for up to 9 months. The diluted serum samples were tested three times.

**TABLE 3:** Results of the stability test

Temp	Reaction at preservation time (h)^*a*^					
	0	1	3	6	9	12
18–25°C (RT)	+++	+++	++	++	+	–

a Values shown are from three replicates.

### Accuracy of rE2-based ICTSs.

The accuracy of the colloidal gold rE2-based ICTSs was evaluated using 136 diluted serum samples (1:100) collected from pig farms (Henan, China) that were compared with the results obtained with the IDEXX CSFV antibody test kit. Overall, 95.6% (128/136) of the samples were positive with both the rE2-based ICTSs and IDEXX CSFV antibody test kit (Table 2), indicating good consistency. The result of the IDEXX CSFV antibody test kit was positive when the blocking rate was >40%. Moreover, the rE2-based ICTSs are relatively simple to use and rapid, and thus more suitable for analysis of serum samples.

### Detection of E2 Abs in sera of rabbits immunized against LAV.

**FIG 4:**
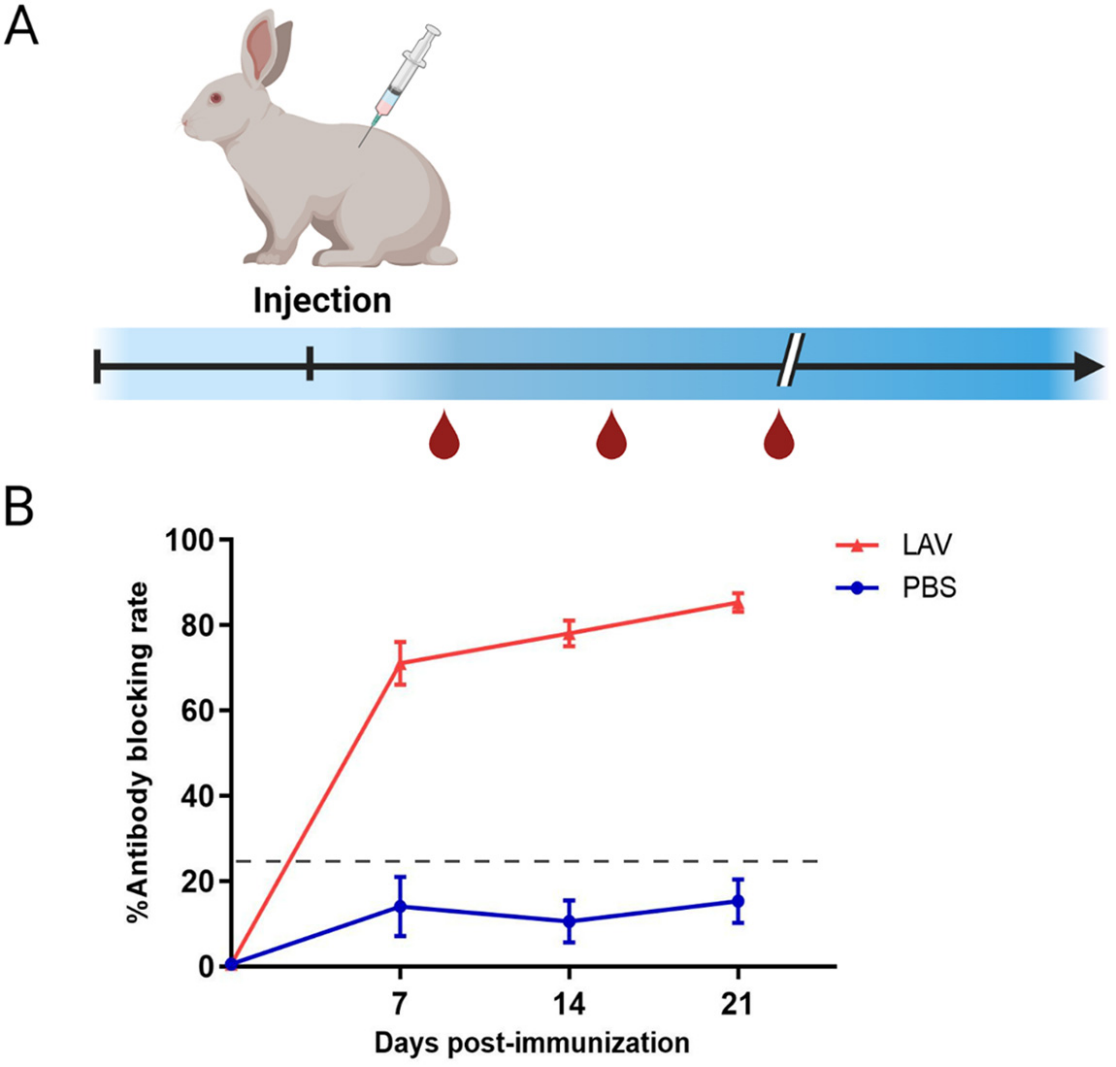
Anti-E2 Abs in serum of vaccinated and challenged rabbits. (A) Rabbit immunization program. (B) Trend graph of blocking rates of vaccinated rabbit serum analyzed with the IDEXX ELISA kit. Horizontal and vertical bars show means ± standard deviations (SD).

Serum samples from rabbits immunized against LAV were used to evaluate the rE2 strips for Ab detection. New Zealand White rabbits (*n* = 4/group) were vaccinated with LAV once (Fig. 4A), and E2-specific Abs were induced as early as 7 days postimmunization, with an average blocking rate of 71%, and animals sustained a high Ab response (*P < *0.001). E2-specific Abs were not detected in the negative control rabbits (Fig. 4B). The results with the rE2 ICTSs were consistent with the increasing trend of Abs detected with a commercial ELISA kit (Table 4), suggesting that the rE2-based ICTSs are suitable for detection of Abs against CSFV in serum samples from rabbits immunized with a LAV.

**TABLE 4:** Sensitivity of rE2-based ICTSs to anti-LAV rabbit serum

Rabbit serum	No. of positive ICTSs	% positive^*a*^ by IDEXX ELISA	Sensitivity^*b*^
LAV-0	0/3	0	–
LAV-7	3/3	75	++
LAV-14	3/3	80	+++
LAV-21	3/3	86	+++
PBS-0	0/3	0	–
PBS-7	0/3	33	–
PBS-14	0/3	25	–
PBS-21	0/3	22	–

a The blocking rate (percent positive) is the mean of three (Intra-assay variability) replicates.

b −, the sensitivity of the strip was negative; ++, the sensitivity of the strip was medium positive; +++, the sensitivity of the strip was strongly positive.

### Evaluation of rE2 strips for detection of MDAs in serum samples from piglets.

In total, 78 serum samples were collected from piglets aged 30 to 40 days. All sera were tested separately with the rE2 ICTSs and IDEXX CSFV antibody test kit. The results were consistent for 92.3% (72/78) of the samples (Table 2), demonstrating that the rE2-based ICTSs can rapidly detect MDAs.

### Evaluation of the rE2 strips for detection of E2 Abs induced with two vaccination strategies.

**FIG 5:**
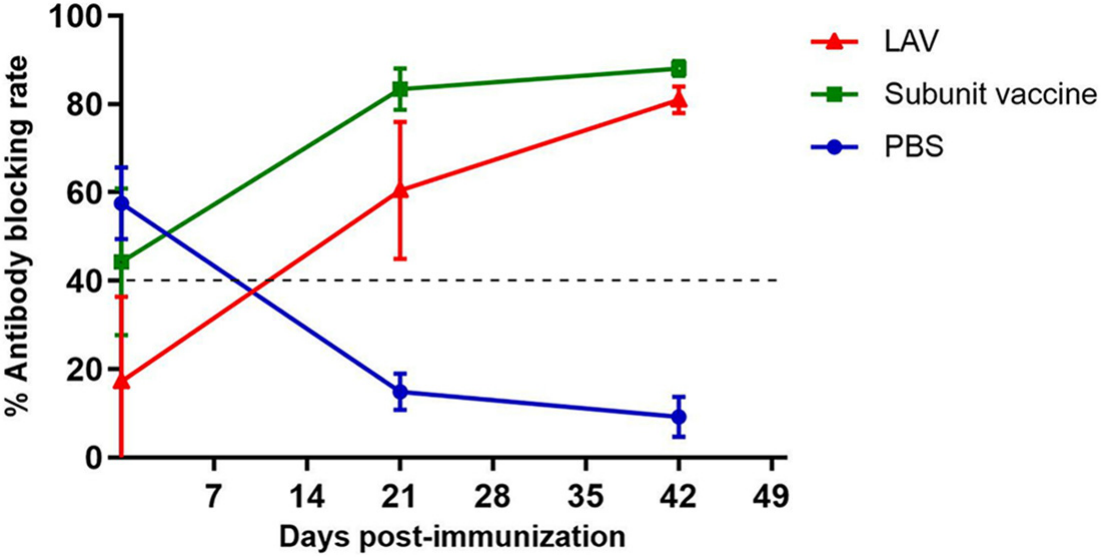
Anti-E2 Abs in serum of pigs inoculated with two different commercial vaccines. Trend graph shows blocking rates of vaccinated pig sera analyzed with the IDEXX ELISA kit. LAV, commercial live attenuated vaccine; subunit vaccine, commercial subunit vaccine. Horizontal and vertical bars show means ± SD.

The performance of the colloidal gold rE2 test strips was evaluated with serum of pigs vaccinated with the recombinant E2 subunit vaccine or live attenuated vaccine. The titers of anti-E2 Abs were measured in serum samples collected from pigs at 0, 21, and 42 days postvaccination. The results showed that Ab levels increased with immunization duration and reached a maximum level after a second immunization, with average blocking values of 88% and 81% for the rE2 subunit vaccine and LAV, respectively. As expected, the serum samples from the negative control pigs were negative with both tests (Fig. 5). In these experiments, the serum samples were also tested with a commercial ELISA kit. The results with the rE2 strip were consistent with those of the IDEXX CSFV antibody test kit (Table 5), thereby confirming that the improved rE2-based ICTSs can be used to detect Ab levels in pig herds with different immunological backgrounds.

**TABLE 5:** Sensitivity of rE2-based ICTSs with different swine serum samples

No.	Serum background	Day^*a*^	No. of positive ICTSs	% positive^*b*^ by IDEXX ELISA	Total
1	LAV immunized	0	3/4	17	4
2	LAV immunized	21	3/4	60	4
3	LAV immunized	42	4/4	81	4
4	E2 vaccine immunized	0	2/4	44	4
5	E2 vaccine immunized	21	4/4	83	4
6	E2 vaccine immunized	42	4/4	88	4
7	Infected	74	4/4	92	4

a Day postvaccination when tested.

b The percent blocking rate is the mean of four replicates (intra-assay variability).

## DISCUSSION

CSFV remains a major challenge to the swine industry. Conventional LAVs can protect animals from viral infection. However, the challenge of controlling the transmission of CSFV remains unresolved due to difficulties with the differentiation of infected and vaccinated animals (DIVA) and interference of MDAs (20, 21). Hence, it is crucial to develop novel marker vaccines based on the E2 protein to eliminate CSFV (22, 23). Currently, several commercially available subunit vaccines can potentially protect against CSFV, although novel and improved diagnostic methods are needed to better monitor Abs against CSFV in immunized animals.

A variety of CSFV diagnostic methods are available for detection of Abs, including commercial ELISA kits against the major antigenic E2 protein, indirect immunofluorescence assays, virus neutralization tests, Western blotting analysis, and colloidal gold ICTSs. Compared to other detection methods, colloidal gold ICTSs are relatively simple to use, rapid, portable, and can be used for high-throughput serological diagnosis (24, 25). Gold nanoparticles (AuNPs), carbon nano-onions, single-walled carbon nanotubes, and chitosan are highly sensitive label-free immunosensors for the detection of bioactive molecules. AuNPs, which are the most extensively used, are biocompatible and have large surface-to-volume ratios and unique electronic properties. AuNPs are often utilized in the form of biofunctionalized particles that can be fabricated via encapsulation in a coating of biological material, such as protein, DNA, or lectin (26). In this study, the rE2 protein was purified from rice seed extract and separated by SDS-PAGE to ensure purity. Anti-CSFV swine serum specifically recognized the rE2 protein, as confirmed by Western blot analysis. The rE2 protein has been proposed as a potential vaccine target against CSFV. Plant-specific posttranslational modifications can facilitate the production of biosimilars and complex recombinant proteins with enhanced function and efficacy (27). Considering the advantages of colloidal gold immunochromatography based on plant-derived E2 as a diagnostic antigen, a colloidal gold rE2-based ICTS was established for rapid detection of CSFV Abs.

The quality of the rE2-based ICTSs is particularly important in field applications. A stable, good-quality homogeneous colloidal gold solution can be obtained (28). Following optimization of the parameters, clear bright red gold solution was obtained. The wavelength of the colloidal gold-labeled rE2 particles was ~550 nm, indicating that the average size of the colloidal gold-labeled rE2 protein was ~44 nm, and thus suitable for use as a gold-labeled probe. If too large or not uniform, the gold particles tend to precipitate, leading to false-positive results (29).

Serum samples were collected to establish the Ab detection assay. Recent studies have reported that pathogen-specific IgA, IgM, and IgG Abs can be detected in oral fluid and nasal swabs collected from various domestic animal species (30). However, little is known about the accuracy and reliance of these samples for monitoring Abs. Testing with serum samples from swine infected with several important viruses showed that the rE2-based ICTSs were highly sensitive and specific. The results of the rE2-based ICTSs and commercial ELISA kit were highly similar, indicating that the rE2-based ICTSs can quickly and conveniently monitor the development of CSFV Abs under different vaccine backgrounds. For measurement of MDA titers, the similarity between the results of the rE2-based ICTSs and commercial ELISA kit was 92.3.0%, suggesting that the rE2-based ICTSs can be used for early detection of Abs against CSFV and in formulating a rational immunization protocol at an earlier time point. In addition, the rE2 strips were also used to evaluate the serum of different animal species (rabbits and pigs) immunized with a LAV or E2 subunit vaccine.

Previous studies have reported the development of diagnostic assays based on the E2 protein expressed in E. coli or insect cell systems. This is the first report of ICTSs based on the E2 protein derived from a plant. As an alternative method, the proposed rE2-based ICTSs avoid the low immunogenicity of antigens expressed by eukaryotic systems and possible contamination of BVDV from fetal bovine serum in cell culture. Moreover, the antigen derived from the transgenic rice system largely reduces cost; thus, the reagent is more economically suitable for clinical use (31).

In conclusion, a plant-derived E2 antigen was used to develop rE2-based ICTSs as an inexpensive, safe, and contamination-free alternative to current assays. Because the amount of rice-derived protein used to prepare the ICTSs is extremely low (6 μg of rE2 protein produces 500 strips), there is no concern that transgenic rice will compete for resources of growing rice for food. The rE2-based ICTSs can be used to monitor vaccine immunity and early detection of MDAs as a rapid diagnostic tool for detection of Abs against CSFV within 5 to 10 min. These findings also illustrate the potential of plant systems for the diagnosis of other pathogens.

## MATERIALS AND METHODS

### Reagents, instruments, and kits.

Gold(III) chloride trihydrate, staphylococcal protein A (SPA), bovine serum albumin (BSA), and ovalbumin were purchased from Sigma-Aldrich (St. Louis, MO, USA). Nitrocellulose (NC) membranes, glass fiber filter membranes, absorbent pads, and ultrapure water were obtained from EMD Millipore (Billerica, MA, USA). All other reagents and solvents were analytical grade or higher quality. A microplate reader was purchased from Bio-Rad Laboratories (Hercules, CA, USA). An XYZ biostrip dispenser, CM 4000 cutter, and TSR3000 membrane strip reader were all purchased from Biodot, Inc. (Irving, CA, USA). A classic swine fever virus antibody test kit was purchased from IDEXX Laboratories, Inc. (Westbrook, ME, USA).

### Sera and rE2 protein for validation of rE2-based ICTSs.

CSFV-positive serum was purchased from the China Institute of Veterinary Drug Control (catalog number S0760905-2; Beijing, China). To optimize and assess the performance of the rE2-based ICTSs, a wide range of clinical serum samples were tested, including CSFV-positive sera (*n *= 10), CSFV-negative sera (*n *= 10), PCV2-positive sera (*n *= 4), PRRSV-positive sera (*n *= 4), FMDV-positive sera (*n *= 4), BVDV-positive sera (*n *= 3), and ASFV-positive sera (*n *= 3). Serum samples (136 from adult pigs immunized with a LAV and 78 from unvaccinated 30-day-old piglets) collected from local pig farms were provided by the Key Laboratory of Animal Immunology (Henan Academy of Agricultural Sciences, Zhengzhou, China). The E2 gene, which encodes the ectodomain of CSFV E2 (GenBank AAK21202.1, residues 1 to 342), was optimized with a rice codon bias (unpublished data). The rE2 protein of CSFV was expressed in rice endosperm as previously described (32) and then purified by ion-exchange chromatography and hydrophobic interaction chromatography.

### Preparation of rE2-based colloidal gold solution.

A solution of colloidal gold was prepared as previously described (33), with slight modifications, and adjusted to pH 8.0 with 0.2 M K_2_CO_3_. Then, 10 μL of rE2 protein (1.2 mg/mL) was diluted 2-fold in double-distilled water (DDW) and mixed with 125 μL of colloidal gold solution for 5 min at RT to determine the most appropriate protein concentration for conjugation. Subsequently, 125 μL of 10% NaCl solution was added and a color change was observed. The color of the reaction changed from bright red to blue as the concentration of the rE2 protein decreased. The optimal concentration of colloidal gold-labeled rE2 is the minimum concentration that retains the red color of the solution.

An appropriate volume of 2.0 mg/mL of the rE2 protein solution in DDW was added dropwise to 50 mL of colloidal gold solution (pH 8.0) for 30 min at RT. Then, 5 mL of 10% BSA was added. After 10 min, the mixture was centrifuged at 13,000 × *g* for 30 min at 4°C. The supernatant was carefully discarded and the precipitant was resuspended in 5 mL of gold suspension buffer. Finally, 5 mL of a gold-labeled rE2 probe was obtained and stored at 4°C for later use.

### Assembly of rE2-based ICTSs.

The rE2-based ICTSs were assembled from three pads (absorbent pad, 30 by 1.8 cm; conjugate pad, 30 by 0.8 cm; sample pad, 30 by 1.5 cm) and an NC membrane (30 by 1.5 cm; HF13502S25; EMD Millipore Corporation) on a semirigid polyethylene sheet. The conjugate pad was sprayed with colloidal gold-labeled rE2 protein using a XYZ biostrip dispenser and heated in an oven for 1 h at 42°C. The sample pad was soaked with treatment fluid (20 mM Na_2_B_4_O_7_, 1% BSA) and dried for 4 h at 42°C. Then, SPA and pig polyclonal anti-CSFV serum were spotted on the test and control lines of the NC membrane, respectively. The distance between the two lines was 0.45 cm. After spotting, the membrane was dried for 4 h at 42°C. Finally, the assembled semirigid polyethylene sheet was cut into strips (0.27 by 5 cm), which were vacuum sealed in plastic bags containing silica as moisture absorbent and stored at 4°C.

### Principle and result readout.

**FIG 6:**
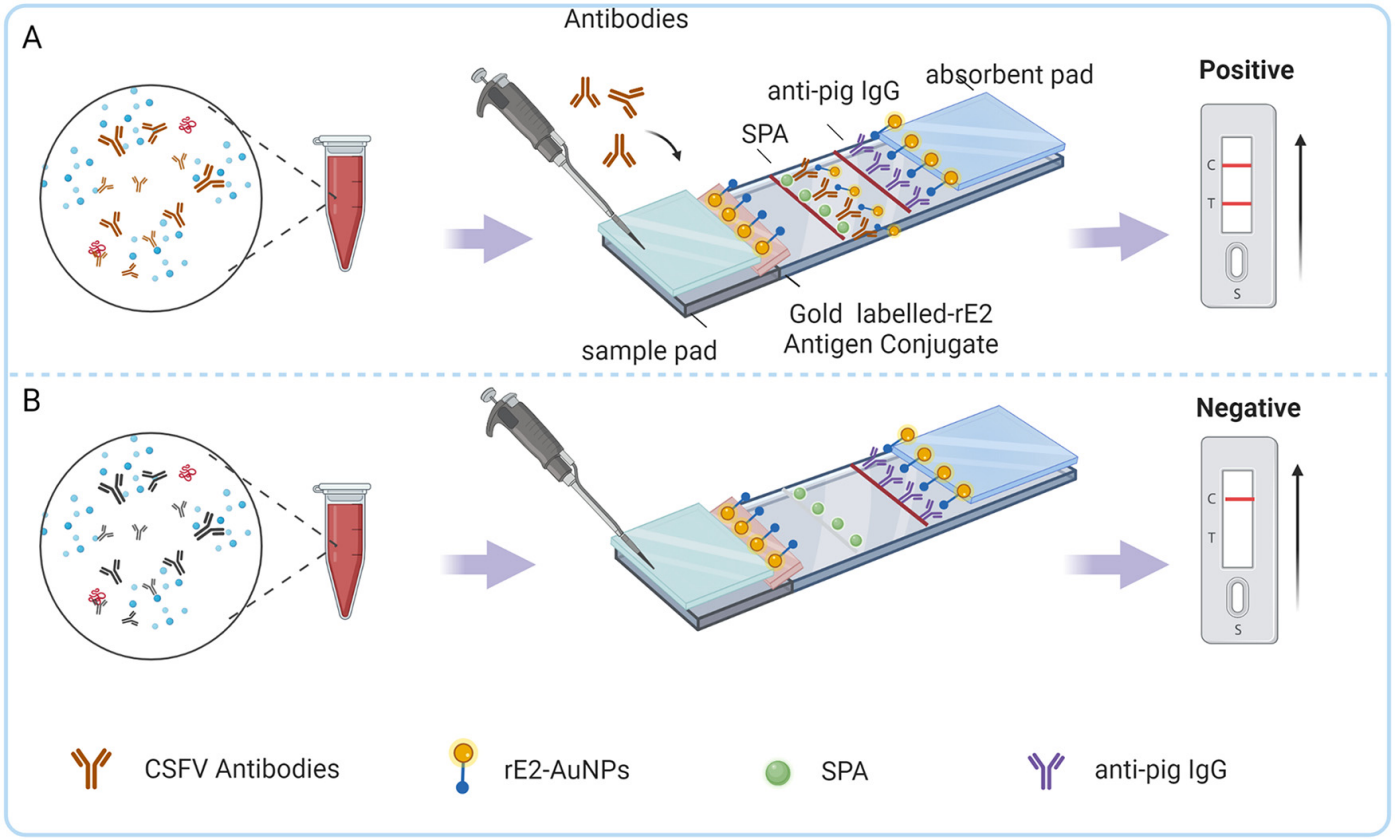
Structure of the rE2-based ICTSs and result readout. (A) For the CSFV-positive serum samples, the gold-rE2 conjugate forms a complex with anti-E2 IgG before binding to SPA, which combines with the IgG Fc domain of the antigen-Ab complex. As a result, the T line with SPA turns red. (B) For a negative serum sample, serum IgG is only combined with anti-pig IgG on the control line, with no color on the test line. C, control line, anti-pig IgG; T, test line, SPA.

Serum was added to the sample pad and moved forward along the NC membrane by capillary action. Once the serum reached the conjugate pad containing E2 Abs, the protein on the gold-rE2 conjugate pad reacted with the corresponding Ab and formed antigen-Ab complexes. Then, IgG trapped by SPA on the test line was visualized as a red band (Fig. 6A). Regardless of whether the tested serum contains E2 Abs, the control line is always colored red, indicating that the material and procedure are correct, while the lack of a red band on the control line indicates that the rE2-based ICTS is invalid (Fig. 6B).

The serum was diluted 100-fold with normal saline, and 100 μL of the diluted sample was added dropwise to the sample pad. The test result was considered positive if the control line appeared red within 5 to 10 min at RT and negative if the control line, but not the test line, contained a red band.

### Performance of rE2-based ICTSs.

The performance of the rE2-based ICTSs was assessed by measuring their sensitivity, specificity, and stability. First, the CSFV-positive serum was diluted with the sample solution from 1:100 to 1:256,000. As described in the section “Principle and result readout,” each dilution was used as an independent sample and the results were determined within 5 to 10 min. Then, the specificity and cross-reactivity of the rE2-based ICTSs were tested with different serum samples, including CSFV-positive sera (*n *= 10), CSFV-negative sera (*n *= 10), PCV2-positive sera (*n *= 4), PRRSV-positive sera (*n *= 4), FMDV-positive sera (*n *= 4), BVDV-positive sera (*n *= 3), and ASFV-positive sera (*n *= 3). Each test was conducted in triplicate. To assess the stability, the specificity and sensitivity of ICTSs that had been vacuum sealed at RT were measured at 0, 1, 3, 6, 9, and 12 months.

### Production of E2 Abs in rabbits immunized with the C strain vaccine.

To effectively apply the prepared ICTSs in clinical practice, rabbits were immunized with an LAV. Three-month-old New Zealand White rabbits (*n* = 4) were purchased from the Laboratory Animal Center of Zhengzhou University. After a 7-day acclimation period, each rabbit was intravenously injected with 1 mL of commercial C strain LAV only once. The ICTSs were used to detect Abs in serum separated on days 0, 7, 14, 21, and 24 and compared to the results obtained with the IDEXX CSFV antibody test kit, which was used in accordance with the manufacturer’s instructions.

### Detection of CSFV Abs in pig serum samples.

Serum samples (136 from adult pigs immunized with a LAV and 78 from unvaccinated 30-day-old piglets) were collected from local pig farms. The colloidal gold rE2-based ICTSs produced in this study and the IDEXX CSFV antibody test kit were used to detect Abs against CSFV. The coincidence rate of rE2 ICTSs was compared to that of the commercial ELISA kit. The kappa values of the rE2 ICTSs corresponding to the IDEXX CSFV antibody test kit for detection of Abs in 136 serum samples from adult pigs and in 78 sample from unvaccinated 30-day-old piglets were 0.530 (*P < *0.05) and 0.256 (*P < *0.05), respectively.

### Application of rE2 strips to test serum samples from pigs immunized with two different commercial vaccines.

Eight piglets (a mixture of males and females; age, 30 days) were randomly divided into two groups of four each and vaccinated followed by a second immunization 4 weeks later. Then, serum samples were collected to assess the immunization status of the piglets vaccinated with the LAV and subunit vaccine. Serum Abs were tested with the colloidal gold rE2-based ICTS and an IDEXX CSFV antibody test kit on days 0, 21, and 42. Detection with the rE2 ICTSs was performed as described above. The experiment was repeated at least three times.
